# Lipid Rafts and Clathrin Cooperate in the Internalization of PrP^C^ in Epithelial FRT Cells

**DOI:** 10.1371/journal.pone.0005829

**Published:** 2009-06-08

**Authors:** Daniela Sarnataro, Anna Caputo, Philippe Casanova, Claudia Puri, Simona Paladino, Simona S. Tivodar, Vincenza Campana, Carlo Tacchetti, Chiara Zurzolo

**Affiliations:** 1 Dipartimento di Biologia e Patologia Cellulare e Molecolare, Università degli Studi di Napoli “Federico II”, Napoli, Italy; 2 Unité de Trafic Membranaire et Pathogénèse, Institut Pasteur, Paris, France; 3 FIRC Institute of Molecular Oncology Foundation (IFOM), Milano, Italy; 4 CEINGE Biotecnologie Avanzate s.c.a.r.l., Napoli, Italy; 5 MicroscoBio Research Center, Università di Genova, Genova, Italy; 6 Dipartimento di Medicina Sperimentale, Università di Genova, Genova, Italy; Iowa State University, United States of America

## Abstract

**Background:**

The cellular prion protein (PrP^C^) plays a key role in the pathogenesis of Transmissible Spongiform Encephalopathies in which the protein undergoes post-translational conversion to the infectious form (PrP^Sc^). Although endocytosis appears to be required for this conversion, the mechanism of PrP^C^ internalization is still debated, as caveolae/raft- and clathrin-dependent processes have all been reported to be involved.

**Methodology/Principal Findings:**

We have investigated the mechanism of PrP^C^ endocytosis in Fischer Rat Thyroid (FRT) cells, which lack caveolin-1 (cav-1) and caveolae, and in FRT/cav-1 cells which form functional caveolae. We show that PrP^C^ internalization requires activated Cdc-42 and is sensitive to cholesterol depletion but not to cav-1 expression suggesting a role for rafts but not for caveolae in PrP^C^ endocytosis. PrP^C^ internalization is also affected by knock down of clathrin and by the expression of dominant negative Eps15 and Dynamin 2 mutants, indicating the involvement of a clathrin-dependent pathway. Notably, PrP^C^ co-immunoprecipitates with clathrin and remains associated with detergent-insoluble microdomains during internalization thus indicating that PrP^C^ can enter the cell via multiple pathways and that rafts and clathrin cooperate in its internalization.

**Conclusions/Significance:**

These findings are of particular interest if we consider that the internalization route/s undertaken by PrP^C^ can be crucial for the ability of different prion strains to infect and to replicate in different cell lines.

## Introduction

Prion diseases are fatal neurodegenerative disorders which are often characterized by a cerebral accumulation of a protease-resistant, misfolded isoform of the prion protein, PrP^Sc^ (scrapie PrP), which derives from the glycosylphosphadidyl inositol (GPI)-anchored cellular isoform PrP^C^ (cellular PrP) [1]–[3]. Compared to PrP^C^, PrP^Sc^ contains an increased number of β-sheet structures, is partially proteinase K-resistant and aggregates and accumulates in the brain [4]. The mechanisms involved in PrP^C^ to PrP^Sc^ conversion are unknown and controversy exists regarding the precise subcellular localization of this event. Both PrP^C^ and PrP^Sc^ have been localized to the plasma membrane and have been shown to undergo endocytosis [5], [6] and this appears to be required both for prion infection and conversion [7]–[12].

Internalization of molecules can occur through the classical, clathrin-mediated pathway for which many molecular components are known [13] and/or via non-classical clathrin-independent routes [14]–[16]. Numerous clathrin-independent pathways are emerging [17]; among these the raft-dependent route can be subdivided into caveolae-dependent and caveolae-independent pathways [18]. Caveolae are membrane invaginations considered to be specialized raft domains, which originate from the oligomerization of their integral coat proteins, the caveolins [19], [20]. They are involved in the uptake of cholera toxin (CTxB), viruses [21]–[23], as well as in the internalization of cross-linked GPI-anchored proteins (GPI-APs)[21], [24] and transmembrane receptors like TGFβ and EGFR [25], [26]. CTxB, tetanus toxin and non cross-linked GPI-anchored proteins are also internalized by less well defined mechanisms involving membrane microdomains known as “lipid rafts”, “detergent resistant microdomains (DRMs) or “caveolae-like domains” (CLDs), which have similar lipid composition to caveolae but lack cav-1 [27]–[29]. However, clathrin-dependent and -independent pathways of internalization may not be as distinct as previously thought [30], [31]. Indeed, lipid rafts have also been implicated in the control of clathrin-mediated internalization of some receptors, such as the BCR (B cell receptor). Interestingly, this receptor can also be internalized by these microdomains independently of clathrin. The observation that the BCR can be endocytosed by both clathrin- and raft-dependent mechanisms may appear paradoxical if one considers that prototypical clathrin-coated pits internalized receptors are not enriched in lipid rafts [32], [33]. Nonetheless, recent reports suggest a connection between mechanisms regulating cell signalling and endocytosis [30], [34], [35]. Indeed, Puri and colleagues have demonstrated that the EGFR-internalizing clathrin-coated pits can assemble within lipid rafts which could represent the cellular sites to coordinate EGFR signalling and internalization [34]. Specifically, lipid rafts might act as platforms that spatially link the signalling machinery with clathrin to regulate the internalization process of specific molecules.

One class of molecules that seem to have access to many of these different pathways are the GPI-anchored proteins (GPI-APs). Non cross-linked GPI-APs are constitutively internalized through a pathway that is independent of clathrin and Dynamin (Dyn) and dependent on rafts and Cdc-42 [29]. However when they are cross-linked they are internalized via caveolae [24], [36], [37] but they can also enter the clathrin-dependent pathway when interacting with transmembrane proteins possessing a clathrin-coated pit internalization signal [37].

Consistent with these findings, the GPI-AP PrP^C^ has been shown to be internalized by either clathrin-dependent [38]–[41], or raft-mediated pathways [42], depending upon the cell types and the different techniques utilized. Furthermore, although there is no direct evidence for caveolae-dependent internalization, the presence of PrP^C^ in caveolae-like domains [8], [9] and its colocalization with cav-1 [12], [43] suggested an involvement of caveolae in PrP^C^ endocytosis.

Understanding the endocytic itinerary of PrP^C^ is fundamental, since data in the literature indicate that both general inhibition of endocytosis [8], [12] and/or a direct modification of the internalization route of PrP^C^, by replacing the GPI anchor with a transmembrane sequence containing a coated pits localization motif [9], affect both the infection and the conversion processes [3], [44].

Using a combined approach of immunofluorescence, electron microscopy and biochemistry we have followed and characterized the internalization of mouse PrP (moPrP) transfected in FRT (Fischer Rat Thyroid) cells that do not express cav-1 and do not have caveolae (FRT-PrP^C^), as well as in FRT cav-1 cells (FRT-PrP^C^/cav-1) which form caveolae [45].

We report that PrP^C^ internalization is delayed by interfering with either the clathrin-dependent or the raft-mediated pathway but is not affected by caveolin expression. We also found that PrP^C^ is in DRMs during the internalization period and co-immunoprecipitates with clathrin from DRMs fractions. Overall our data indicate that lipid rafts and clathrin cooperate for PrP^C^ internalization. Consistent with this, PrP^C^ internalization is completely blocked only under simultaneous impairment of both clathrin and lipid rafts.

## Results

### Endocytosis of PrP^C^ is dependent on cholesterol but independent of cav-1

Although PrP^C^ has been previously reported to be in caveolae [43] or caveolae-like domains [8], [9], there is no direct experimental evidence demonstrating or disproving a role for caveolae in PrP^C^ internalization.

**Figure 1:**
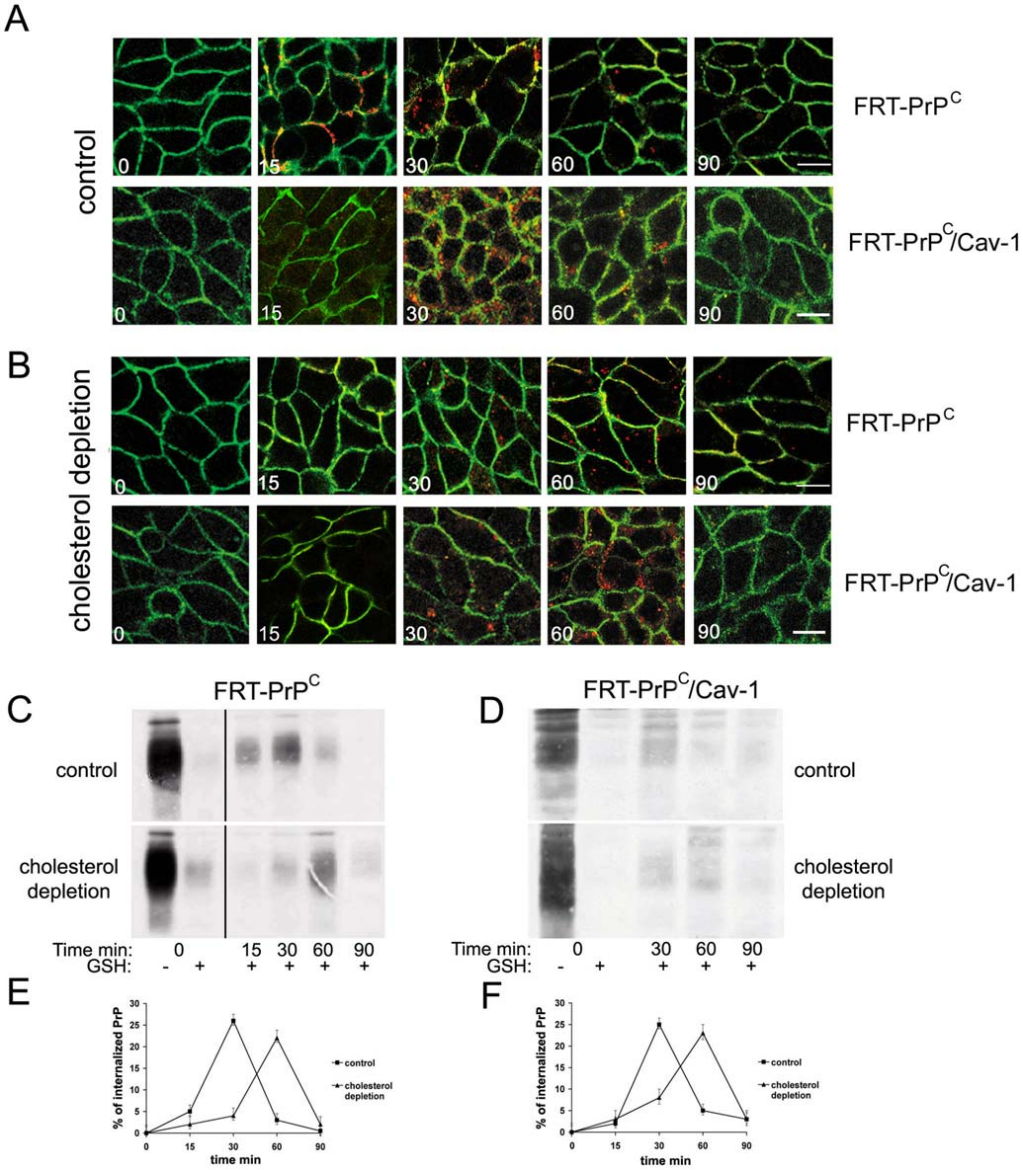
The kinetic of PrP^C^ internalization is delayed by cholesterol depletion but it is not affected by caveolin expression. A) and B) FRT-PrP^C^ cells or FRT-PrP^C^/cav-1 grown on transwell filters were subjected to indirect immunofluorescence under control (A) and cholesterol depletion conditions (B). Cells were incubated for 30 min with αPrP antibody (SAF32) at 4°C on the basolateral side of the filters and subsequently, warmed up to 37°C to allow PrP^C^ internalization for the indicated times. Surface PrP^C^ is labelled in green, while internalized PrP^C^ is in red (see Materials and Methods for experimental procedure). Bar: 10 µm. FRT-PrP^C^ (C) or FRT-PrP^C^/cav-1 cells (D), grown under control and cholesterol depletion conditions on transwell filters, were subjected to a biotinylation based endocytic assay (see Materials and Methods). PrP^C^ was immunoprecipitated with SAF32 antibody and the surface and the internalized PrP^C^ were revealed by western blotting with streptavidin-HRP. E) and F) The data from four independent experiments performed in C) and D) respectively were quantified using NIH-image for MacIntosh, and plotted in the graphs. The amount of biotinylated internalized PrP^C^ was expressed as a percentage of the amount of PrP^C^ on the surface at 4°C, which we set as 100%. Error bars are indicated. Note that the different smear corresponding to PrP^C^ between Figure 1C and 1D is due to differences in the gel migration and not to differences in PrP^C^ glycosylation pattern between cells expressing caveolin or not (see Figure S1B).

FRT cells stably transfected with mouse PrP (moPrP) cDNA (FRT-PrP^C^ cells) have previously been used to characterize the exocytic pathway of PrP^C^ and PrP^C^ mutants [46]–[48]. Furthermore, these cells represent an ideal system to test whether caveolin and caveolae are involved in PrP internalization because they do not express cav-1 and caveolae but form functional caveolae following transfection of cav-1 [45]. Therefore, to directly assess the possible role for caveolae in PrP^C^ internalization, we stably transfected FRT-PrP^C^ cells with a cDNA encoding for human cav-1. Consistent with previous reports [45], transfection of cav-1 was sufficient to promote *de novo* formation of caveolae in FRT-PrP^C^ cells and at steady-state cav-1 was found to be associated with smooth flask-shaped invaginations at the plasma membrane (Figure S1A *a,* 15 nm gold). Interestingly, double-immunolocalization on ultrathin cryosections with αPrP and αcav-1 antibodies (respectively 10 and 15 nm gold), revealed colocalization of the two proteins on caveolae stemming from the cell surface (*a*). In order to characterize the endocytic pathway followed by PrP^C^, we developed an indirect immunofluorescence assay in cells grown in polarized conditions, which allowed us to distinguish between internalized and surface protein (Figure 1A, Materials and Methods). By confocal microscopy, we observed that PrP^C^ begins to enter the cells after 15 minutes of incubation at 37°C, and shows a peak of internalization after 30 min (Figure 1A, FRT-PrP^C^). This slow kinetic of endocytosis is compatible with a caveolae or caveolae-like mediated internalization pathway [49]; however in FRT-PrP^C^/cav-1 cells PrP^C^ was internalized with similar kinetics to wild type cells (compare the panels of Figure 1A FRT-PrP^C^ and FRT-PrP^C^/cav-1), showing a peak of internalization at 30 min. Therefore, these data show that cav-1 and caveolae did not affect PrP^C^ endocytic trafficking. Noteworthy, in line with the EM data, immunofluorescence analysis revealed that although at steady-state PrP^C^ and cav-1 are both preferentially localized at the cell surface (Figure S1C), once internalized they were never found to share the same intracellular distribution neither after 30 min nor after 60 min (the same is for 15 min, not shown) (Figure S1D).

Interestingly, morphometric analysis of EM data (Figure S1A and see below) indicated that PrP^C^ is found in clathrin-coated pits in both FRT-PrP^C^ and FRT-PrP^C^/cav-1 cells in similar amounts indicating that transfection of cav-1 does not alter the plasma membrane distribution of PrP^C^.

Altogether these data indicate that cav-1 transfection did not affect the internalization levels or kinetics of PrP^C^ that we had observed in the absence of caveolae demonstrating that although PrP^C^ can be found in caveolae it is not endocytosed via those structures.

Having excluded a role for caveolae we next analyzed the role of rafts which have been implicated in the uptake of different GPI-anchored proteins and of PrP^C^ itself [12], [42]. Because raft-mediated internalization is sensitive to drugs that interfere with the homeostasis of cholesterol in membranes, we depleted cells of cholesterol by mevinolin/methyl-β-cyclodextrin using conditions that lowered cholesterol to ∼50% of normal levels and that have been shown to impair PrP^C^ raft association [46], [50], [51], (Materials and Methods). Interestingly, under these conditions the kinetics of PrP^C^ endocytosis was decreased and a peak of internalization occurred only after 60 min of chase both in FRT-PrP^C^ and FRT-PrP^C^/cav-1 cells (Figure 1B).

In order to quantify these data and to avoid the use of full IgG molecules, which have been previously shown to cross-link GPI-anchored proteins and eventually change their internalization pathway [36], [40], [52], we used a biochemical internalization assay based on the use of a reducible biotin reagent [53] (Figure 1C, D). FRT and FRT-cav1 cells expressing PrP^C^ were grown on filters and then selectively biotinylated on the basolateral surface with NHS-LC-SS-Biotin and chased at 37°C for the indicated times to allow internalization. By addition of glutathione, which reduces biotin at the cell surface but does not have access to the internalized proteins, we could discriminate between the cell surface and the internalized PrP^C^ fractions, at the different chase times. Interestingly, the amount of internalized PrP^C^ was not changed by lowering cholesterol levels. Indeed, about 20% of surface PrP^C^ was internalized in both control and cholesterol depleted cells (Figure 1C, D and E, F). However, in agreement with the immunofluorescence data, the biotinylation assay showed that the kinetics of PrP^C^ internalization was delayed by cholesterol depletion resulting in a peak of internalization at 60 minutes in contrast to 30 minutes for control cells (Figure 1C, D and E, F). These results (Figure 1 and S1A, D) further exclude a role for caveolae in PrP^C^ uptake and support the involvement of a cholesterol-dependent mechanism suggesting a role for lipid rafts in this process.

Importantly, in order to exclude pleiotropic effects of cholesterol depletion on membrane function, including inhibition of clathrin-dependent internalization, previously reported in other cell lines by acute cholesterol depletion [54], [55], we could show that cholesterol depletion did not impair internalization of Transferrin (Tfr), a marker of the clathrin-dependent endocytosis (Figure S2A, B and Supporting information for fluorescence quantification).

### Internalization of PrP^C^ depends on Cdc-42 activity, Dyn 2 and Eps15-AP2 binding

The results presented above, that PrP^C^ endocytosis was slowed down but not completely blocked by cholesterol depletion, could be explained by two possibilities. Either the molecular mechanism involved in the PrP^C^ endocytic pathway is not solely dependent on membrane rafts, or under partial cholesterol depletion conditions the raft-dependent endocytic pathway still functions, albeit less efficiently.

**Figure 2:**
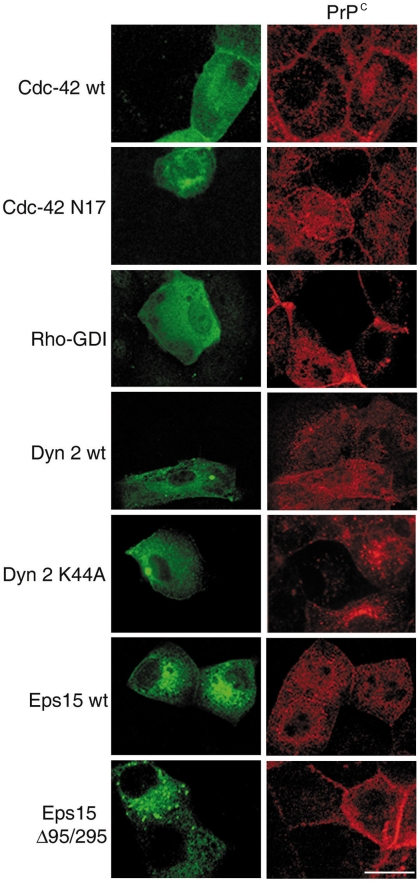
PrP^C^ internalization is impaired by transfection of Cdc-42, Dyn 2, Eps15 GFP-tagged dominant-negative mutants and of Rho-GDI (His_6_-tagged form). FRT-PrP^C^ cells were transiently trasfected with the His6-tagged form of Rho-GDI or with the wild-type (wt) or dominant negative mutant isoforms of GFP-tagged Eps15 (Eps Δ95/295), Cdc-42 (Cdc-42 N17) or Dynamin 2 (Dyn 2 K44A). Cells were then challenged with SAF32 antibody binding to PrP^C^ at 4°C, warmed for 30 min at 37°C and processed for immunofluorescence. Rho-GDI was revealed by an anti-His6-tag Ab. Bar: 10 µm. Note that PrP^C^ internalization is affected by overexpression of dominant negative isoforms of Cdc-42, Dyn 2 and Eps15 but not by the wild-type forms.

Since PrP^C^ has been shown to be internalized both *via* a raft-dependent or a clathrin-dependent pathway [38]–[40], [56] depending on the cell lines and the experimental methods utilized, we decided to dissect the endocytic pathway of PrP^C^ by identifying the specific molecular factors involved in its uptake. To this aim we tested whether the transient expression of dominant-negative mutants of known molecules involved in different endocytic pathways would perturb PrP^C^ internalization. Specifically, we expressed the GFP-tagged versions of the dominant-negative isoform of Cdc-42, Dyn 2 and Eps15 and employed a fluorescent microscopy-based internalization assay to follow PrP^C^ (Figure 2). As positive and negative controls we used fluorescently labelled Tfr or CTxB as markers of the clathrin- or raft-dependent routes respectively (Figure S3).

First, we analyzed the effect of the dominant-negative, GFP-tagged Cdc-42 isoform (Cdc-42 N17). Cdc-42 is a component of the Rho family of GTPases and has been shown to be specifically involved in the clathrin-independent endocytosis of GPI-anchored proteins [29] the FGF2 receptor [57] and the Helicobacter pylori VacA cytotoxin [58]. By quantification of the fluorescence (see Material and Methods) we found that the expression of Cdc-42 N17, but not the wild-type GFP-linked form, reduces the amount of internalized PrP^C^ *per* cell (Figure 2 and Table 1). As expected, Cdc-42 N17 had only a slight effect on the internalization of Tfr (Table 1, and Figure S4) thus indicating that in FRT cells PrP^C^ might be internalized via a raft-mediated pathway dependent upon activation of Cdc-42.

**Table 1:** Internalization of PrP^C^ is dependent on Cdc-42, Rho, Dyn 2, Eps15 and clathrin.

**% of internalization**	**Cdc42 wt**	**Cdc42 N17**	**RhoGDI**	**CHC siRNA**
Tfr	98±0.2	93±1.2	96±2.3	9±1.9
PrP^C^	96±2.2	52±2.0	19±1.4	22±1.3
CTxB	99±1.0	10±1.5	**-**	**-**
**% of internalization**	**Dyn 2 wt**	**Dyn 2 K44A**	**Eps15 wt**	**Eps15 Δ95/295**
Tfr	95±1.5	25±1.4	99±1.0	15±2.5
PrP^C^	98±1.5	30±2.6	90±3.2	34±2.8

**Figure 3:**
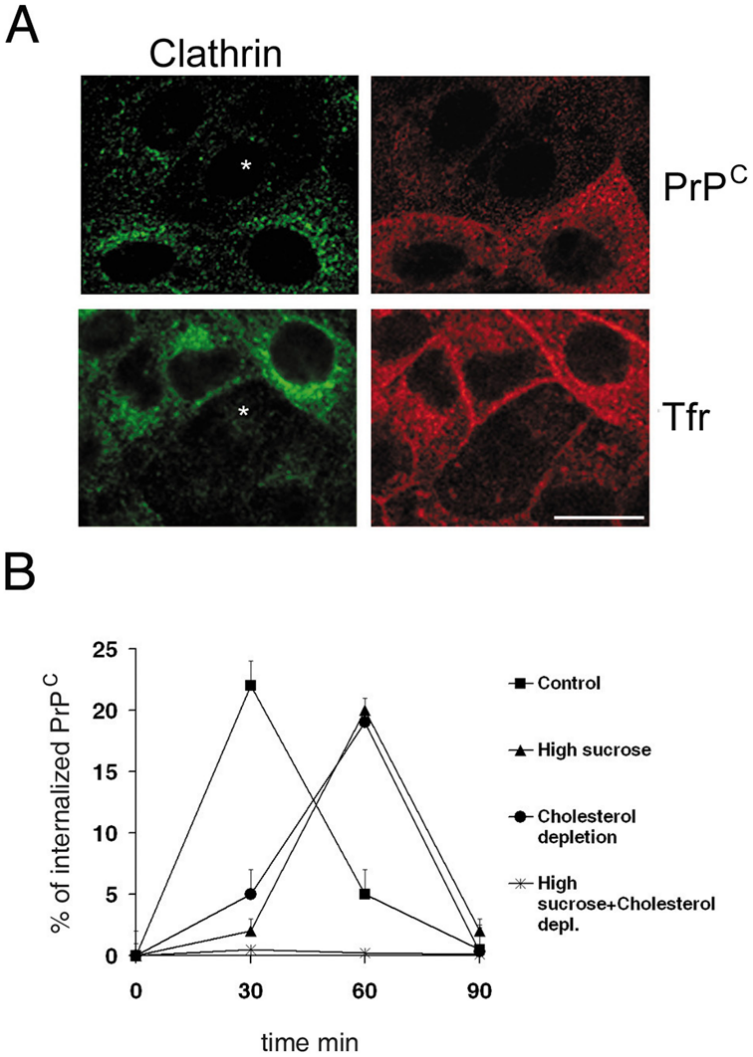
Endocytosis of PrP^C^ is both clathrin and raft-dependent. A) FRT-PrP^C^ cells transfected with siRNA against clathrin heavy chain (CHCsiRNA, asterisks indicate transfected cells), were incubated with Pri308 antibody to PrP^C^ at 4°C, warmed for 30 min at 37°C and processed for immunofluorescence. Clathrin was revealed with a monoclonal αclathrin antibody clone ×22 (ABR bioreagents) and a TRITC-conjugated secondary antibody; PrP^C^ was revealed by cy3-secondary antibody and Tfr was cy3-conjugated. The amount of clathrin and internalized PrP^C^ in each cell was analyzed by Image J software from three independent experiments using 4–5 coverslips for each CHCsiRNA independent transfection. Bar: 10 µm. B) FRT-PrP^C^ cells, grown in control and cholesterol depletion conditions on transwell filters were subjected to cell surface biotinylation with NH-SS-biotin at 4°C on the basolateral side of the filters. The cells were then warmed at 37°C to allow PrP^C^ internalization for indicated times in medium containing or not high sucrose. Glutathione was used to reduce the residual proteins not internalized from the plasma membrane. PrP^C^ was immunoprecipitated with Pri308 or SAF32 antibody and the surface and the internalized PrP^C^ were revealed by western blotting with streptavidin-HRP. Data were quantified as in Figure 1E, F. Error bars are indicated.

Cells transfected with the different GFP-tagged wt or dominant negative isoforms, with His6-tagged Rho-GDI or siRNA against clathrin heavy chain, were analyzed by confocal microscopy and the results were quantified with Image J software by selecting different optical sections (z stacks, see Materials and Methods) in which we measured the intensity of fluorescence (IF)/unit of area. Data are from experiments repeated three times using 4–5 coverslips for each transfection. The amount of internalized Tfr, PrP^C^ and CTxB (in 30 min of internalization) in the cells transfected with the wild-type or dominant negative GFP-tagged isoforms was expressed as a percentage of the amount in untrasfected cells, which we set as 100% (±S.D. are indicated). The percentages of internalized PrP^C^ and Tfr in siRNA cells for CHC were calculated by measuring the ratio between the average of the values of IF/unit of area in siRNA treated cells (see asterisks in the Figure 3A) and the average values of IF/area in untrasfected cells.

To further characterize this pathway we analyzed the effect of Rho*-*GDI expression on PrP^C^ and Tfr internalization in these cells. Rho-GDI overexpression locks Cdc-42 in a GDP-inactive state resulting in its removal from the membrane-associated pool. As expected from the results on the effect of mutant Cdc-42, the internalization of PrP^C^ was also perturbed in FRT cells transfected with a His_6_-tagged form of Rho-GDI (Figure 2 and Table 1). However, we did not find any effect of the Rho-GDI on Tfr internalization (Figure S3 and Table 1). This is in agreement with data showing that clathrin-mediated endocytosis is mainly regulated by Rho and Rac which, opposite to Cdc-42, need to be locked in the active state to block internalization [59]. Because raft-mediated internalization can be either Dynamin-dependent or -independent [60], [61], we decided to examine the involvement of Dyn 2 by using the Dyn 2 K44A dominant-negative mutant, which has been extensively used as a tool to block the fission of endocytic intermediates [62]–[64]. Consistent with previous reports, transient transfection of a GFP-tagged Dyn 2 K44A cDNA impaired the clathrin-dependent internalization of Tfr [65] (Figure S3 and Table 1). Similarly, PrP^C^ endocytosis was also perturbed under these conditions. In contrast, in control cells expressing wild-type Dyn 2, both PrP^C^ and Tfr were internalized normally (Figure 2, Table 1 and Figure S3). This result could be explained either by an involvement of a raft-mediated Dyn 2–dependent internalization of PrP^C^ or by the involvement of a clathrin-mediated pathway in PrP^C^ endocytosis. In order to test this latter hypothesis we used the isoform of Eps15 mutated in the AP2 binding domain (Eps15 Δ95/295), that has been demonstrated to selectively inhibit clathrin-mediated endocytosis [26], [66], and followed both PrP^C^ and Tfr internalization. We found that the transient transfection of the dominant-negative mutant but not of the wild-type Eps15 decreased the endocytosis of both PrP^C^ and Tfr (Figure 2, Table 1 and Figure S3), thus supporting the involvement of clathrin in PrP^C^ internalization. As an alternative approach we transiently transfected in FRT cells a siRNA directed against the clathrin heavy chain (CHCsiRNA)[67] which resulted in an efficient reduction of detectable clathrin as well as reduced internalization of PrP^C^ and Tfr in transfected cells (Figure 3A and Table 1). Collectively, the results obtained using both siRNA and dominant-negative mutants show that PrP^C^ internalization is cholesterol-dependent and is regulated by molecular factors involved in both raft- and clathrin-dependent pathways.

### PrP^C^ internalization is blocked only by the combined use of hypertonic medium and cholesterol depleting drugs

**Figure 4:**
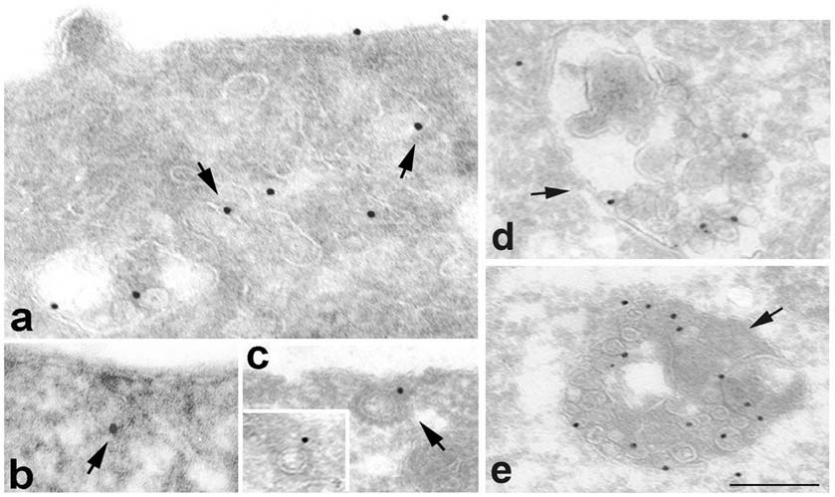
PrP^C^ internalization by electron microscopy analysis. (*a–c*): Immunogold labeling of PrP^C^ on ultrathin cryosections of FRT cells under steady-state culture conditions reveals that PrP^C^ (15 nm gold) is localized within both smooth and morphologically identified clathrin-coated pits; *(d–e)* FRT cells have been incubated 30 min on ice in the presence of antibody against PrP. After 30 min incubation at 37°C the cells were fixed and processed for cryo ultramicrotomy. Ultrathin cryosections were immunogold labeled using protein A-gold (15 nm). As shown also in the absence of a possible surface cross-linking of the two antibodies due to the protein A, PrP^C^ reaches the endosomal compartments. Bar: *a* = 235 nm; *b* = 245 nm; *c* = 215 nm; *d,e* = 263 nm. Arrows point to the different intracellular compartments labelled with αPrP antibodies. Morphometric analysis on 1800 total gold particles revealed n = 0 PrP gold particles in flask-shaped structures and n = 25 in clathrin-coated pits.

The data presented above support roles for both clathrin- and a raft- mediated pathways in PrP^C^ internalization. In order to quantitatively define their respective involvement we performed the biotinylation-based endocytic assay described above utilizing a hypertonic milieu that causes the disruption of clathrin lattices [68], in combination or not with cholesterol depletion (Figure 3B). Intriguingly, disruption of clathrin lattices affected PrP^C^ internalization in a manner similar to cholesterol depletion. Specifically, both treatments delayed the kinetics of endocytosis by 30 minutes without altering the total amount of the internalized protein (Figure 3B). These results suggest that both clathrin- and raft-dependent pathways are utilized for PrP^C^ internalization; therefore, a testable prediction of this hypothesis is that it should be possible to completely block PrP^C^ uptake by pharmacologically poisoning both pathways simultaneously. To this end we measured PrP^C^ internalization in cells subjected to a combined treatment of cholesterol depletion and exposure to a hypertonic milieu. Consistent with the stated hypothesis, under these conditions PrP^C^ uptake was completely blocked (Figure 3B) suggesting that clathrin-dependent and cholesterol/raft-dependent pathways account for all the routes of PrP^C^ internalization. Importantly, under these conditions both the staining of a basolateral protein marker Antigen 35/40 kDa as well as the values of the transepithelial resistance (TER) of the monolayers were unaffected (Figure S5), thus ruling out pleiotropic effects of the combined treatment on the plasma membrane. The hypothesis of the involvement of both clathrin- and raft- dependent pathways in the endocytosis of PrP^C^, was also strengthened by the analysis of its distribution at steady-state and during internalization by Electron Microscopy (Figure 4). Immunogold labeling on ultrathin cryosections of FRT-PrP^C^ cells at steady-state shows that PrP^C^ (15 nm) localizes mainly in smooth areas and invaginations of the plasma membrane (Figure 4*a, b*) but also in morphologically identified clathrin-coated pits and vesicles (Figure 4*c*) and after 30 min at 37°C in the endolysosomal compartment (*d, e*).

### Clathrin and lipid rafts cooperate for PrP^C^ internalization

The utilization by PrP^C^ of both routes of internalization raises the question as to how these two pathways are regulated and how they control PrP^C^ endocytosis. Whether they are completely separate or overlapping, or whether they interact. Indeed, there are three possible models to explain our previous data: either PrP^C^ moves laterally out of lipid rafts before undergoing endocytosis by “classical” clathrin-dependent pathway as previously proposed [40], or clathrin directly associates with PrP^C^-containing rafts as shown in the case of the BCR [30] and CTxB [35] or PrP^C^ is internalized by both the clathrin- and raft-mediated pathways as independent spatially segregated mechanisms.

**Figure 5:**
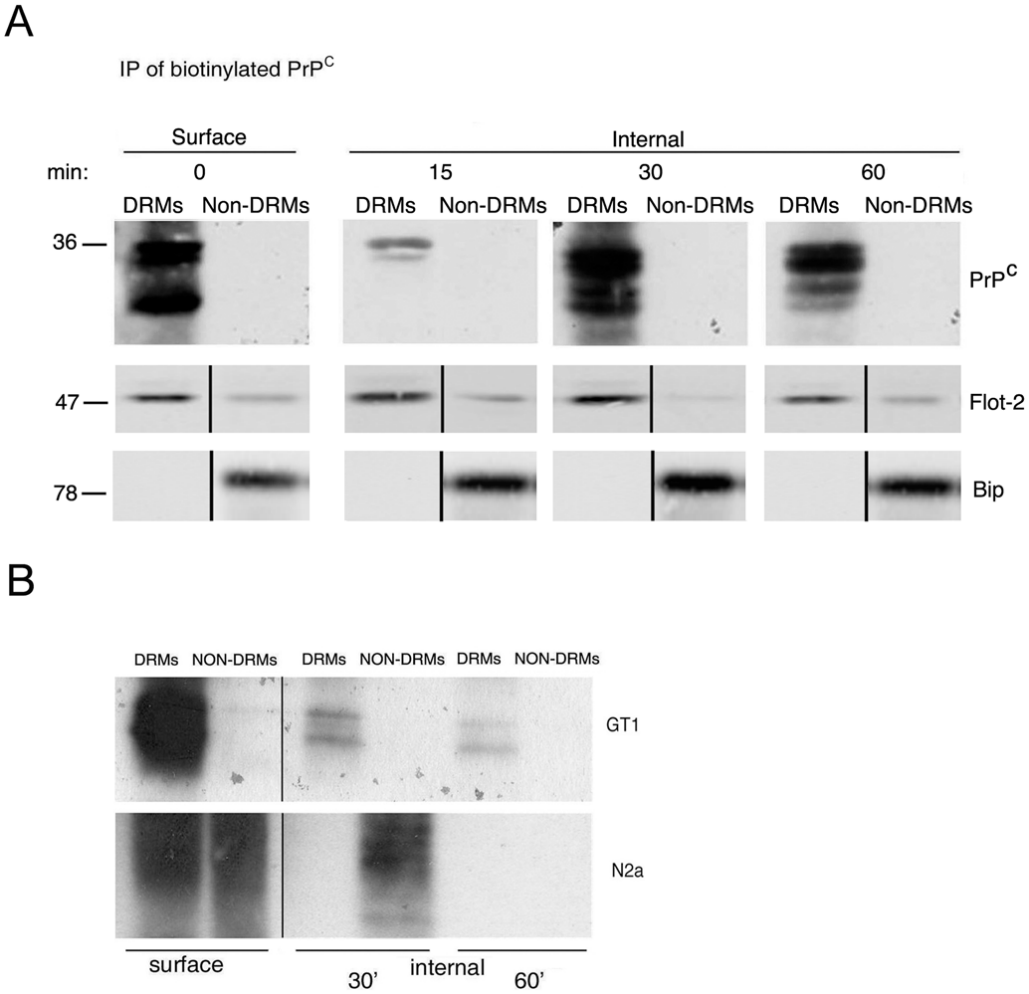
Internalization of PrP^C^ occurs in DRMs. A) FRT-PrP^C^ cells grown for 4 days on filters and incubated with disulfide-linked biotin at 4°C, were either solubilized immediately (surface) or first incubated for 15, 30 and 60 min at 37°C to allow internalization and then subjected to biotin stripping by a reductive cleavage and then solubilized (internal). The cell lysates were subjected to a “Two Step” OptiPrep™ density gradient to separate DRM (fractions 4–5) from non-DRM fractions (6–12). Fraction 5 and 11 were immunoprecipitated with αPrP SAF32 antibody and biotinylated PrP^C^ was revealed with streptavidin-HRP. To test the efficiency of fractionation, before PrP^C^ immunoprecipitation, an aliquot of fraction 5 and 11 was immunoblotted for Flotillin-2 (Flot-2 as raft marker, [82]) and Bip/Grp78 (as non-raft marker, [80]). B) GT-1 and N2a cells grown for 4 days on dishes were incubated with disulfide-linked biotin at 4°C and were either solubilized immediately (surface) or first incubated for 30 and 60 min at 37°C to allow internalization. They were then subjected to biotin stripping by a reductive cleavage and solubilized (internal). The cells were subjected to a “Two Step” OptiPrep™ density gradient to separate DRM (fractions 4–5) from non-DRM fractions (6–12). Fraction 5 (DRMs) and 11 (non- DRMs) were immunoprecipitated with αPrP SAF32 antibody and revealed with streptavidin-HRP.

To discriminate among these three mechanisms, we analyzed the association between PrP^C^ and DRMs both on the cell surface and during internalization (Figure 5A). After treatment with disulfide-linked biotin, cells were incubated at 37°C to allow endocytosis and were subjected to density gradient purification in order to discern PrP^C^ raft-association. We found that PrP^C^ was present in DRMs and that it remained associated with them during the entire internalization time, suggesting that it does not exit “raft domains” to be internalized via the clathrin-coated pits. This result suggested that PrP^C^ internalization via clathrin pathway was occurring in raft domains. Notably, we obtained the same results in the neuronal cell model of GT-1 [69] where PrP^C^ associates with DRMs both on the surface and after internalization (Figure 5B). Interestingly, this is in contrast with observations in N2a cells where it is reported that the internalized pool of PrP^C^ is not associated to DRMs [40]. Interestingly, we could confirm these results using our experimental conditions in N2a cells, showing that internalized PrP^C^ exits from DRMs (Figure 5B). These data support the hypothesis that PrP^C^ can enter different cell lines using different internalization routes [42]. Since internalized PrP^C^ remains associated with rafts both in FRT and GT-1 cells, we assessed whether PrP^C^ was associated with rafts and clathrin simultaneously.

**Figure 6:**
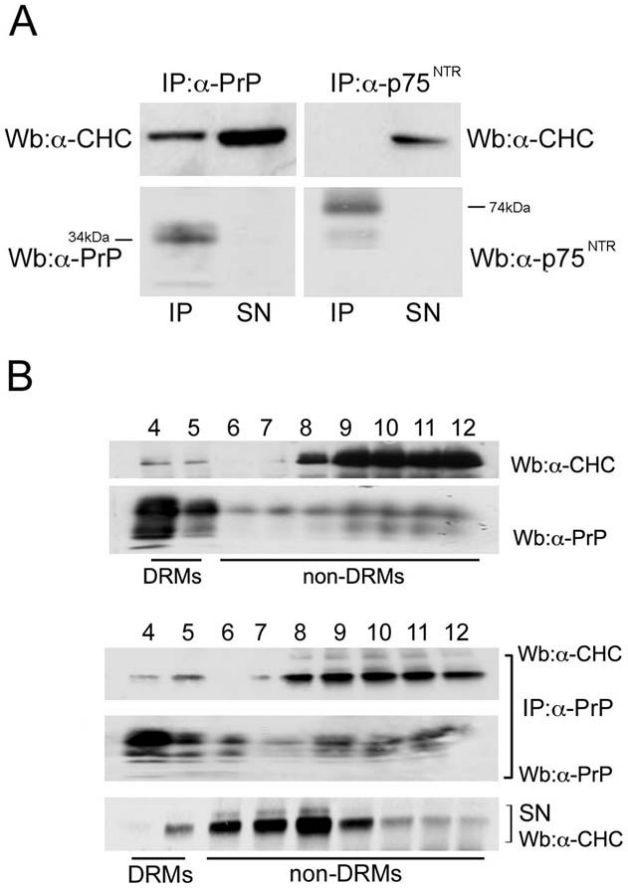
PrP^C^ and clathrin co-immunoprecipitate both in DRM and non-DRM fractions. A) FRT-PrP^C^ cells grown on filters were lysed, immunoprecipitated (IP) by αPrP antibody (SAF32) or αp75^NTR^ antibody (as negative control), and revealed using: αCHC (TD.1) or αPrP and αp75^NTR^ antibody as indicated. The supernatants (SN) of the immunoprecipitates were tested by western blot to verify the efficiency of the coimmunoprecipitation. B) Filter grown FRT-PrP^C^ cells were lysed and run on OptiPrep™ gradient to separate DRM from non-DRM fractions. The relative distribution of CHC and PrP^C^ in the fractions (upper panel) was tested by western blot of an aliquot of gradient fractions with specific antibodies. The remaining amount of each fraction (bottom panel) was immunoprecipitated with αPrP antibody SAF32 and revealed by αCHC TD.1 antibody. Supernatants (SN) of the immunoprecipitation (IP) are shown in the bottom panel.

To test this hypothesis we performed co-immunoprecipitation assays between PrP^C^ and the heavy chain of clathrin (CHC) and found that the two proteins co-immunoprecipitate (Figure 6A). As negative control of the procedure we used p75^NTR^ [the neurotrophin receptor known to be internalized via a clathrin-independent pathway in neurons [70]], that indeed does not coimmunoprecipitate with clathrin.

Then, in order to understand whether PrP^C^ and clathrin co-immunoprecipitate inside or outside DRMs, we first analyzed the distribution of the two proteins on a OptiPrep™ density gradient (Figure 6B, top panel) and then performed co-immunoprecipitation assays from the DRM and non-DRM fractions of the gradients. As expected PrP^C^ and clathrin showed an opposite enrichment, respectively in DRM and non-DRM fractions of the gradients (Figure 6B, top panel).

However, we found that clathrin co-immunoprecipitated with PrP^C^ both from the DRM and non-DRM fractions (Figure 6B, lower panel). Because we have shown that all internalized PrP^C^ is in DRMs (Figure 5A), the portion of PrP^C^ that co-immunoprecipitates with CHC in DRMs is likely to derive from the plasma membrane and internalized fraction. Thus, these data suggest that a cooperative pathway exists between rafts and clathrin for PrP^C^ internalization.

On the contrary the amount of PrP^C^ that co-immunoprecipitates with clathrin in non-DRMs is possibly associated with intracellular membranes, likely with the Golgi apparatus where the majority of PrP^C^ resides in FRT cells [47].

## Discussion

Understanding the molecular mechanism that controls PrP^C^ endocytic pathway is of particular interest because there is evidence that the initial steps in the PrP^C^-PrP^Sc^ conversion might take place on the cell surface or during/after internalization of PrP^C^ [2], [5].

Current evidences in the literature indicate that PrP^C^ internalization can occur via a clathrin-, caveolae-, and raft dependent pathways [38]–[44].

The discrepancies between these data could be due to differences between mammalian and chicken PrP^C^ sequences (∼30% homology) alternatively used in the different studies and/or to the overexpression of exogenous PrP^C^ molecules in neuroblastoma cells. Alternatively they could derive from the different cells and the various internalization assay utilized [38]–[43]. Furthermore it is not clear whether PrP^C^ can undertake multiple internalization pathways in the same cell, and the molecular factors controlling its endocytosis have not been uncovered. This could be very relevant for both understanding the prion pathogenesis, as stated above, but also to reveal the basis of the different prion strains ability to infect and replicate in different cells [71].

In order to characterize the molecular factors involved in PrP^C^ endocytosis and to discriminate among clathrin, caveolae- and raft- dependent mechanisms in PrP^C^ internalization we have used the FRT cells as model system. These cells have been well characterized for GPI-protein and PrP^C^ exocytosis [47], [72] and do not possess caveolae, but form *de novo* caveolae upon cav-1 transfection [45]. We show here that PrP^C^ is localized both in coated and in smooth areas of the plasma membrane (Figure 4) and in line with other evidence [73], it is internalized in late endosomal/lysosomal structures (Figure 4 *d–e*) with a peak of internalization after ∼30 minutes (Figure 1). Based on the circumstantial evidence of its association with caveolae or CLDs [12], [43] it was proposed earlier that PrP^C^ was internalized via a caveolar pathway. By using FRT cells stably transfected with cav-1 we were able to demonstrate that this is not the case in this cell line. Indeed while PrP^C^ co-localizes with cav-1 in caveoale on the surface of FRT-PrP^C^/cav-1 cells (Figure S1) it does not colocalize with cav-1 during endocytosis. Furthermore cav-1 expression does not have any effect on the kinetics of PrP^C^ internalization, which was similar in both FRT-PrP^C^ and FRT-PrP^C^/cav-1 cells (Figure 1).

Having excluded a role for caveolae in PrP^C^ endocytosis we analyzed the role of rafts by assaying the dependence of PrP^C^ internalization on cholesterol.

Interestingly we observed that following cholesterol depletion the internalization of PrP^C^ was delayed, but not completely inhibited. While these results suggest a role for rafts in PrP^C^ internalization they could be explained by two possibilities. Either the molecular mechanism involved in the PrP^C^ endocytic pathway is not solely dependent on membrane rafts, or under partial cholesterol depletion conditions the raft-dependent endocytic pathway still functions, albeit less efficiently.

In order to discriminate between these two possibilities and to characterize the molecular factors involved in PrP^C^ internalization, we used dominant negative forms of different molecules previously shown to be involved in clathrin- and/or raft-dependent endocytosis [29], [64], [74]–[76].

By using the Cdc-42 N17 dominant negative form we found that the internalization of PrP^C^ and CTxB, but not Tfr uptake, were impaired (Figure 2, Table 1, Figure S3 and S4). Combined with the effect of Rho-GDI overexpression which affects PrP^C^ but not Tfr internalization (Figure 2, Table 1 and Figure S3), these data strongly suggest that PrP^C^ is internalized by a raft-dependent mechanism. However, because PrP^C^ endocytosis was also affected by dominant negative Dyn K44A we could not discriminate whether the raft-dependent pathway of PrP^C^ internalization was Dynamin-dependent or whether a clathrin-mediated pathway was also involved. Indeed, Dyn 2 regulates fission of clathrin-coated invaginations and caveolae, but also the formation of uncoated, raft-dependent vesicles in cells that do not express caveolae as in the case of IL2R (Interleukin-2 Receptor) internalization [65]. In order to distinguish between these two hypothesises we utilized the dominant negative form of Eps15 (Eps15 Δ95/295) which has been shown to selectively inhibit clathrin-mediated endocytosis [74]. Interestingly, we found that over-expression of this mutant affected to a similar extent the internalization of PrP^C^ and Tfr (Figure 2 and Figure S3), suggesting that a clathrin-dependent pathway was also participating in PrP^C^ endocytosis. This was further confirmed by the transfection of siRNA for clathrin which affected PrP^C^ internalization (Figure 3A and Table 1).

**Figure 7:**
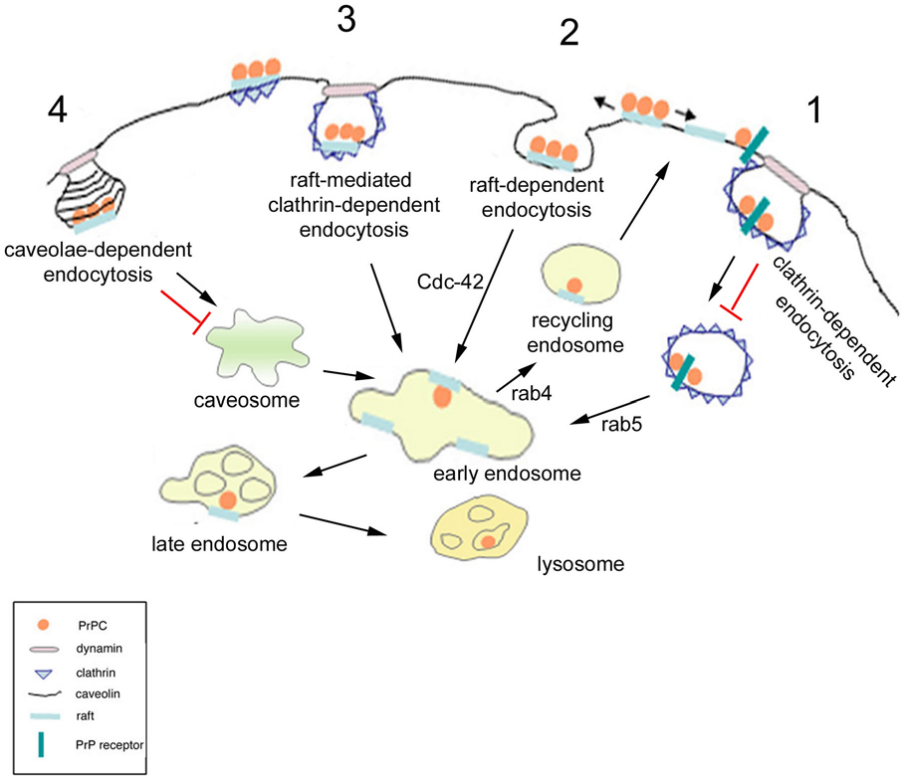
Mechanisms of PrP^C^ internalization in FRT cells. Internalization of molecules can occur throughout many different pathways: the classical, clathrin-mediated pathway (1); the clathrin-independent raft-dependent pathway (2); the raft-mediated clathrin-dependent route (3) and caveolae (4). At the cell surface of FRT cells PrPC is localized in DRMs. As PrPC remains in these domains during its internalization a classical clathrin-dependent pathway (1) seems to be excluded. Conversely PrPC can undertake a rafts dependent pathway (2) and/or rafts might promote its recruitment to clathrin domains inducing its internalization (3). PrPC resides in caveolae at the plasma membrane but is not internalized via the caveolar pathway (4).

The fact that PrP^C^ internalization is dependent on both DRMs and clathrin was also supported by the finding that PrP^C^ endocytosis was completely blocked only when the cells where concomitantly depleted of cholesterol and incubated in a hypertonic milieu. In order to understand whether these two pathways were concomitant or independent from each other we analyzed DRM association during internalization. Noteworthy, our results show that PrP^C^ is in DRMs at the cell surface and remains associated with these domains after 60 minutes of internalization (Figure 5A) when the majority of the protein has been endocytosed (Figure 1A, C, D, E, F) thus indicating that clathrin assembly occurs within these domains. Furthermore, we show that PrP^C^ remains associated to DRMs through all endocytosis also in GT-1 cells (Figure 5B), thus excluding the hypothesis that this was an idiosyncrasy of FRT cells. These data are in agreement with the finding of Ledesma and colleagues who described a raft-mediated mechanism for PrP^C^ internalization in primary neurons [42]. In contrast Sunyach et al., reported that in N2a cells PrP^C^ leaves lipid rafts to enter non-rafts membrane from which it is internalized by a classical clathrin-mediated pathway [40]. Importantly we were able to confirm these results in N2a cells (Figure 5B) thus indicating that the differences observed between N2a, FRT and GT-1 cells were not due to the use of different techniques but likely to cell specific differences. Therefore, taken together our results support the hypothesis that PrP^C^ can be internalized by different mechanisms in different cell types. In addition to this concept our data indicate that PrP^C^ can be internalized by different pathways in the same cell. Indeed, we clearly show that in FRT cells both raft and clathrin are contributing to the efficiency of PrP^C^ uptake. Our data also support the possibility that rafts promote recruitment of PrP^C^ to clathrin domains in a similar fashion to that demonstrated by Abrami et al., [77] and Puri et al., [34]. Indeed it is noteworthy that association with clathrin pits is not mutually exclusive with association to cholesterol containing domains [77]. Earlier studies showing low cholesterol in clathrin vesicles were due to steric hindrance by the clathrin lattice, preventing access of filipin to the vesicles [76]. The cooperation between rafts and clathrin in the internalization of PrP^C^ could also be linked to the signalling function proposed for PrP^C^ [75] which should occur in raft domains [11] and is consistent with the hypothesis proposed for other signalling receptors that lipid rafts could spatially coordinate the signalling machinery while clathrin regulates their internalization [30], [34], [35], [77]. While these data support the role for rafts in the clathrin dependent endocytosis of PrP^C^ our findings also point towards a role for a clathrin-independent, raft- dependent pathway (Figure 7). Furthermore, the fact that depletion of one of these two components (either cholesterol for the raft-mediated pathway or clathrin) is not sufficient to block PrP^C^ internalization, indicates that PrP^C^ is able to enter multiple pathways in order to be endocytosed (Figure 7). The discovery of redundant endocytic pathway for PrP^C^ in the same cell line is particularly relevant if one considers that the internalization route/s undertaken by PrP^C^ can be crucial for the ability of different strains to infect, replicate and induce cell death in different cells. Moreover, the entry in distinct endocytic pathway in response to different stimuli could allow PrP^C^ to exert both the still debated neuroprotective and neurodegenerative functions proposed for this protein.

Further experiments are required to clarify which is the relative contribution of raft-dependent and raft-mediated clathrin-dependent pathway in PrP^C^ internalization.

How rafts and clathrin are reciprocally regulated and what their respective roles are in prion infection and conversion should now be evaluated in infected cells.

## Materials and Methods

### Reagents and antibodies

Cell culture reagents were purchased from Gibco Laboratories (Grand Island, NY). The SAF32 and Pri308 antibodies were from Cayman Chemical (USA). Protein-A-Sepharose was from Pharmacia Diagnostics AB (Uppsala, Sweden). The antibody against cav-1 and His_6_-tag were from Santa Cruz (INC.). Polyclonal αVAMP3 antibody was from Synaptic Systems, Germany. Cholera toxin β subunit (CTxB) Alexa-555-conjugated was from Molecular probes (code: C-34776). Mouse monoclonal antibodies (mAbs) specific for CHC were ×22 from ABR Bioreagents and TD.1 [78]. Cy3- and Alexa-488-conjugated (Tfr) Transferrin were from Invitrogen (Molecular Probes). Monoclonal αp75^NTR^ Ab was a kind gift of Dr. Andrè Le Bivic. Policlonal αBip/Grp78 antibody was from Stressgen Biotech. αFlotillin-2 antibody was from BD Trasduction Laboratories. Methyl-β-cyclodextrin (MβCD), mevinolin and all other reagents were obtained from Sigma Chemical Co. (St Louis, MO).

### Constructs and transfections

FRT cells were stably transfected with a cDNA encoding 3F4 tagged moPrP^C^ with the calcium phosphate procedure, as previously described [53].

The GFP-tagged Cdc-42 wild type or mutant (Cdc-42 N17) were a gift from Mayor S. (National Center for Biological Sciences, UAS-GKVK Campus, India). The GFP-tagged Eps15 wild type or dominant negative mutants (EpsΔ95/295) were described previously [74] and were a gift from A. Dautry-Varsat (Institut Pasteur, Unité de Biologie des Interactions Cellulaires, France). The GFP-tagged Dyn 2 wild type or mutant (K44A) were gifts from MA. McNiven (Mayo Clinic, Rochester, MN; [64]). Rho-GDI was a kind gift from Lamaze C. (Laboratoire "Trafic et Signalisation", CNRS, Institut Curie, Paris, France).

For transient transfection FRT cells were trypsinized and washed three times with serum free RPMI. The cells (1×10^6^) were transfected by electroporation with 15 µg of plasmid encoding the various mutants in a Biorad apparatus (300 V and 500 µF). The cells were resuspended in F12 medium 5% FBS, plated on coverslips and cultured for three days before endocytosis assay.

### Clathrin knockdown by small interfering (siRNA)

The siRNA sequence targeting human clathrin heavy chain (5′-TAA-TCC-AAT-TCG-AAG-ACC- AAT-3′) was a kind gift of Dr. Alice Dautry-Varsat [67]. FRT cells were transfected with 100 pmol of clathrin siRNA following the procedure described above. After three days of culture the cells were washed and used for internalization assay. Clathrin was detected using the antibody clone ×22 from ABR Bioreagents.

### Cell culture, drug treatment and cholesterol determination

FRT cells stably expressing moPrP^C^ were cultured and depleted of cholesterol as previously described [46]. Briefly, FRT cells were plated on dishes or filters and mevinolin (10 µM or 30 µM for 48 h) was added to the cells 24 h after plating in F12 supplemented with 5% delipidated calf serum and mevalonate (200 µM). FRT cells were allowed to grow for another 48 h. MβCD (10 mM) was added to the medium containing 20 mM Hepes, pH 7.5, and 0.2% bovine albumin for 1 h at 37°C to cells pretreated with mevinolin/mevalonate for 47 h.

GT-1 and N2a cells were grown in DMEM (Dulbecco's modified eagle medium) supplemented with 10% FBS.

Cholesterol determination. In order to assay cholesterol levels in the cells before and after treatment with mevinolin/mevalonate and methyl-β-cyclodextrin we used a colorimetric assay. FRT cells grown in the presence or absence of mevinolin/mevalonate were washed twice with PBS, lysed with appropriate lysis buffer and Infinity Cholesterol Reagent (Sigma Chemical Co. ST Louis, Mo, code number 401–25 P) was added to the lysates in the ratio 1∶10 for 5 minutes at 37°C (according to the suggested Sigma protocol number 401). The samples were then measured in a spectrophotometer at 550 nm.

### Internalization assay: fluorescence microscopy and biochemical analysis

Fluorescence Microscopy. Control or cholesterol depleted FRT cells stably expressing PrP^C^ (or PrP^C^ and cav-1), were grown for 4–5 d on filters, washed with PBS, and processed for the endocytic assay. Briefly, the cells were incubated with anti-prion SAF32 (IgG2) antibody (2 µg/ml) 30 min at 4°C (pulse). After Ab binding, the cells were warmed at 37°C to allow PrP^C^ internalization for different indicated times (chase), the monolayers were fixed with paraformaldehyde (PFA) and incubated with the anti-mouse FITC-conjugated secondary antibody to label surface PrP^C^. Before permeabilization with 0.075% saponin we quenched the free binding sites of the FITC-conjugated secondary antibody with a rabbit a-mouse Ab. Internal PrP^C^ was revealed by TRITC-conjugated secondary Ab after permeabilization.

In the case of total cav-1 detection, we used αcav-1 polyclonal primary antibody revealed by a cy5-conjugated secondary antibody after permeabilization. Before cell permeabilization the free binding sites of the rabbit anti-mouse Ab used for quenching the mouse IgG of SAF32 Ab, were saturated by an anti-rabbit Ab.

The percentages of internalization were calculated, using Image J software, by measuring the Intensity of Fluorescence (IF) per unit of area. The fluorescent signal corresponding to the internalized molecules was calculated considering the IF values from the whole z stack excluding the slices corresponding to surface staining. To avoid possible artefacts due to a wide range of expression levels of GFP-dominant negative isoforms in transiently transfected cells, the highest and lowest expressing cells were excluded from the analysis. Colocalization between PrP^C^ and cav-1 was determined in at least 25 cells from four different experiments under control or cholesterol depletion conditions. The analysis was performed by Image J or LSM 510 software and the number of colocalizing pixels was normalized for the total number of internalized PrP^C^ pixels.

Biochemical analysis. Control or cholesterol depleted FRT-PrP^C^ and FRT PrP^C^/cav-1 cells were grown for 4–5 d on filters, washed with PBS, and processed for internalization assay. The cells cooled on ice and biotinylated with NH-SS-Biotin at 4°C [79] on the basolateral side of the filters, were held at 37°C for indicated times. Then residual surface NH-SS biotin was removed by reductive cleavage at 4°C with gluthatione (GSH G4251-1G, from SIGMA). Biotinylated PrP^C^ was immunoprecipitated with SAF32 antibody and revealed by western blotting using streptavidin-HRP conjugated and ECL.

Block of clathrin-dependent internalization was achieved by hypertonic treatment (120 mM NaCl, 12 mM MgSO_4_, 1 mM EDTA, 15 mM sodium acetate, 1% w/v BSA, 100 mM HEPES pH 7.0, 5 mM KCl and 0.4 M sucrose) 30 min before the biochemical assay and during the chase times.

### Purification of surface DRM- and non DRM-associated PrP^C^

Cells grown on filters were incubated with disulfide-linked biotin at 4°C and either solubilized immediately (surface) or first incubated for different times at 37°C to allow internalization. They were then subjected to biotin stripping by a reductive cleavage and then solubilized (internal) in TNE/TX-100 1% buffer (25 mM Tris-HCl [pH 7,5], 150 mM NaCl, 5 mM EDTA, 1%TX-100) on ice. Lysates were scraped from filters, brought to 40% OptiPREP™, and then placed at the bottom of a centrifuge tube. A OptiPrep™ gradient (5–35% TNE, [80], [81]) was layered on top of the lysates. After ultracentrifugation at 20.000 rpm for 4 h at 4°C, one ml fractions (12 fractions in total) were harvested from the top of the gradient. Specifically, starting from the top of the gradient the fraction 5 (representing DRMs) and 11 (non-DRMs) were separately collected, immunoprecipitated with αPrP and revealed with streptavidin-HRP.

### Immunoprecipitation

Immunoprecipitation of PrP^C^ was carried out as previously described [47].

### Immunogold labeling on cryosections and morphometry

FRT-PrP^C^ and FRT-PrP^C^/cav-1 cells were fixed in paraformaldehyde 2%/glutaraldehyde 0.2% in PBS, 2 h at room temperature, scraped off the culture dish, placed in a solution of 12% gelatine in PBS. Small 1 mm^3^ gelatin blocks were embedded with 2.3 M sucrose, overnight, mounted on alluminium pins and frozen in liquid nitrogen. Ultrathin cryosections were immunolabelled with αPrP antibody SAF32 followed by protein A-gold (15 nm, UMC- Utrecht) and or rabbit antibodies against cav-1 (Santacruz) and protein A-gold 10 nm, followed by αPrP and protein A-gold (15 nm).

Alternatively the cells were incubated with αPrP SAF32 for 30 min on ice and then 20 min at 37°C prior fixation. Epon and cryo sections were observed to Philips EM10 or Fei TECNAI 12G2 electron microscopes.

The morphometry analysis on the surface distribution of gold particles was performed counting 1800 and 1917 gold particles over 3612 µm of plasma membrane profiles, in FRT-PrP^C^ and PrP^C^/cav-1, respectively. Among these particles we identified those associate to *bona fide* morphologically identified clathrin-coated pits and caveolae stemming from the cell surface. Only single flask-shaped caveolae clearly associated to the cell surface were counted. We did take into consideration the gold particles associated to organelles identifiable as caveosomes, or clusters of flask-shaped membrane bound structures present underneath the plasma membrane, as we could not define their potential association to the plasma membrane. In the case of FRT-PrP^C^ cells, no caveolae were identified. Data were obtained by comparing two independent experiments.

## Supporting Information

Figure S1 PrP^C^ localizes in caveolae in cav1-transfected FRT cells. A) Double immunolocalization on ultrathin cryosections of PrP^C^ (10 nm gold), and caveolin-1 (15 nm gold), on caveolae stemming from the cell surface (a). Ultrathin sections of FRT-PrP^C^/cav-1 cells, incubated 30 minutes on ice in the presence of α PrP antibody (SAF 32), followed by proteinA-gold (10 nm gold) at 37°C for 20 min, show PrP^C^ localized in morphological identified caveolae at the plasma membrane (b,c). A morphometry analysis of the distribution of gold particles identifying PrP^C^ shows the presence of labeling in flask-shaped structures in the FRT-PrP^C^/cav-1 but not in the FRT-PrP^C^ cells. On the contrary, clathrin-coated pits were found labeled in both type of cells. Arrowheads indicate α PrP gold labelling (10 nm). Bar: a = 238 nm; b = 360 nm; c = 240 nm. Morphometric analysis on 1917 total PrP^C^ gold particles revealed n = 7 in caveolar structures and n = 27 in CCP (clathrin-coated pits, not shown). The remaining 1883 gold particles were distributed on smooth areas of the plasma membrane. PrP^C^ gold particles were counted over 3612 µm of plasma membrane profiles where the number of caveolae and coated pits was comparable (not shown). Note that the percentage of PrP^C^ localized in CCP is similar to wild-type FRT cells (see Figure 4) suggesting that the transfection of caveolin-1 does not affect the surface distribution of PrP^C^. B) Lysates from FRT-PrP^C^ and FRT-PrP^C^/cav-1 cells were immunoblotted with α PrP antibody SAF32. M: mature diglycosylated form, I: immature form and U: unglycosylated form. C) Immunofluorescence of PrP^C^ and cav-1 at steady-state shows a similar distribution on the cell surface of FRT-PrP^C^/cav-1 cells but not after 30 or 60 min of internalization D), suggesting that PrP^C^ endocytosis is not occurring by a caveolae-mediated mechanism (see Methods for experimental procedure).
(8.98 MB TIF)

Figure S2 Internalization of Tfr after cholesterol depletion. A) FRT-PrP^C^ cells were grown on coverslipis and incubated for 30 at 37°C in F12 medium with Tfr Alexa 488-conjugated under control or cholesterol depletion conditions. The cells were then fixed with PFA and incubated with αVAMP3 antibody (a marker of the recycling endosomes [83], after permeabilization with saponin. Single and double immunofluorescences (merge) are shown. As shown in panel A, we found that both in control and cholesterol depleted cells Tfr is internalized and partially colocalizes with VAMP3. We measured the internal intensity of fluorescence (IF)/unit of area in optical sections (Z stacks) comprised between the apical and basal surfaces by LSM 510 confocal microscope. A medial optical section is shown. Bar: 10 µm. B) The amount of internalized Tfr in cholesterol depleted cells was expressed as a percentage of the amount of internalized Tfr in control cells, which we set as 100%. Error bars are indicated. The % of internalized Tfr was determined in at least 25 cells from four different experiments under control or cholesterol depletion conditions. The analysis was performed by Image J software.
(13.57 MB TIF)

Figure S3 Internalization of CTxB and Tfr upon transient transfection of Cdc- 42, Dyn 2, Eps15 GFP-tagged wt or dominant-negative mutants and of Rho-GDI (His6-tagged form). FRT-PrP^C^ cells were transiently transfected with wild-type (wt) or dominant negative mutant isoforms of GFP-tagged Eps15 (Eps Δ95/295), Cdc-42 (Cdc-42 N17) or Dyn 2 (Dyn 2 K44A). His6-tagged form of Rho-GDI was transiently transfected in FRT-PrP^C^ cells that were processed for immunofluorescence as in Figure 2. CTxB was Alexa-555 conjugated and Tfr was cy3-conjugated. Bar: 10 µm. Data were quantified as described in Table 1.
(4.30 MB TIF)

Figure S4 Internalization of Tfr after transient transfection of Cdc-42 wt or N17 mutant. Internalization of Tfr was evaluated after transient transfection of Cdc-42 wt or N17 GFP-tagged isoforms by incubating the cells with cy3-conjugated Tfr internalized for 30 min at 37°C. Bar: 10 µm. Data were quantified as described in Table1.
(5.07 MB TIF)

Figure S5 Analysis of Ag35/40 localization and measurement of transepithelial resistance after cholesterol depletion and hypertonic treatment. A) FRT-PrP^C^ cells were grown on transwell permeable filter supports (Costar), in control or cholesterol depleted conditions and incubated for 30 min at 37°C in control or high sucrose medium. Cells where then stained for the basolateral marker Ag35/40 adding a specific antibody either to the apical or basolateral side of the filters. Samples were analyzed with a Zeiss Laser Scanning Confocal Microscope (LSM 510) equipped with a planapo 63× oil-immersion (NA 1.4) objective lens. Bar: 10 µm. B) Transepithelial resistance (TER) was measured for 7 days after plating 2×10^6^ cells on 24 mm diameter transwell filters in control or cholesterol depletion conditions. After 4 days of culture, only cholesterol depleted cells were incubated for 30 min at 37°C in high sucrose medium and TER was measured by the Millicellers apparatus (Millipore). Note that we process the cells at 4 days of culture, when the TER is maximal and that the combined treatment does not affect the monolayer integrity at this time.
(15.09 MB TIF)
