# Supplementary figures and images for: Lipid Rafts and Clathrin Cooperate in the Internalization of PrPC in Epithelial FRT Cells
Source: PLoS One. 2009 Jun 8;4(6):e5829. doi: 10.1371/journal.pone.0005829 (PMC2688078; doi:10.1371/journal.pone.0005829)

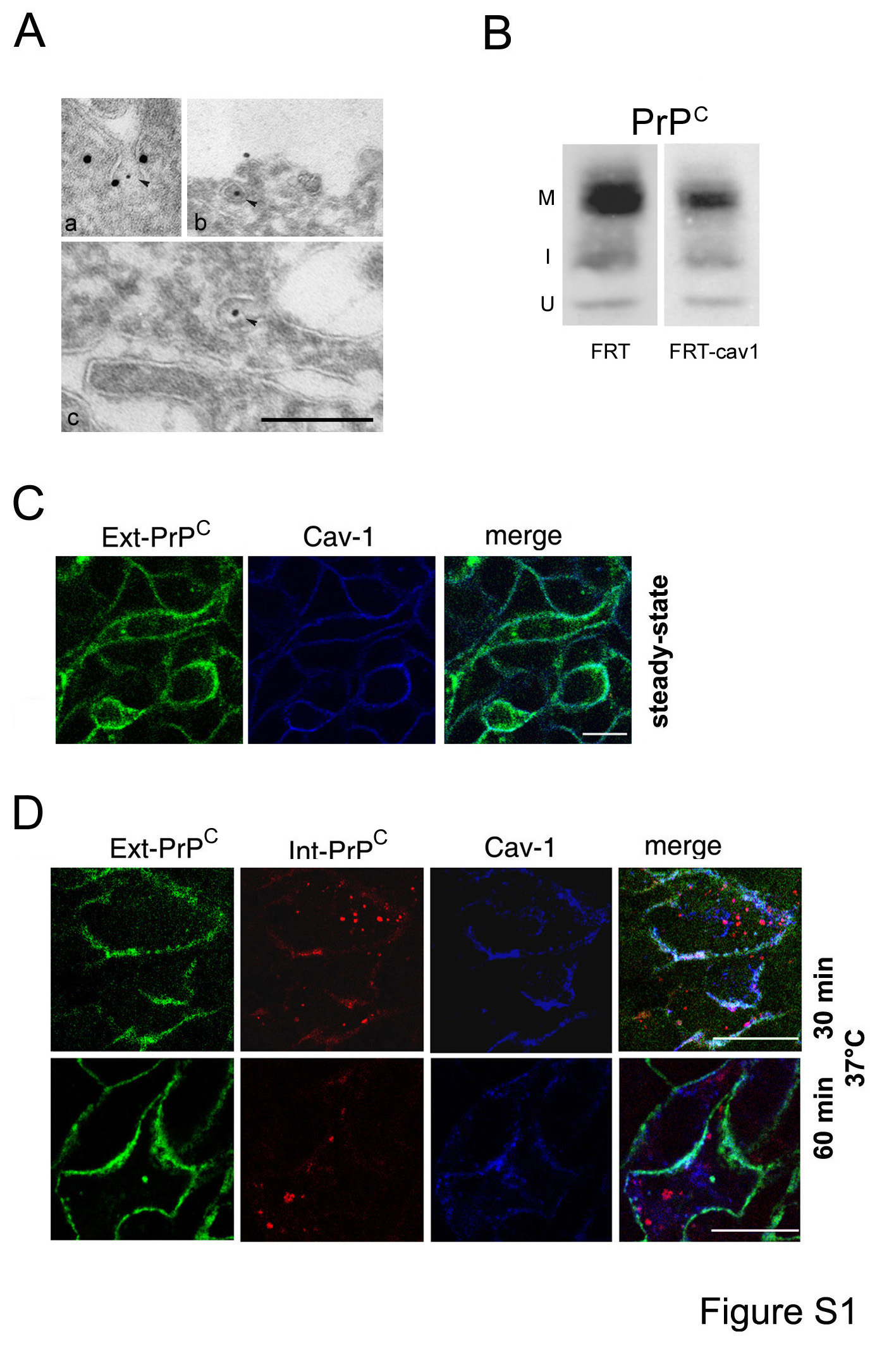

Supplement: Figure S1 — PrPC localizes in caveolae in cav1-transfected FRT cells. A) Double immunolocalization on ultrathin cryosections of PrPC (10 nm gold), and caveolin-1 (15 nm gold), on caveolae stemming from the cell surface (a). Ultrathin sections of FRT-PrPC/cav-1 cells, incubated 30 minutes on ice in the presence of α PrP antibody (SAF 32), followed by proteinA-gold (10 nm gold) at 37°C for 20 min, show PrPC localized in morphological identified caveolae at the plasma membrane (b,c). A morphometry analysis of the distribution of gold particles identifying PrPC shows the presence of labeling in flask-shaped structures in the FRT-PrPC/cav-1 but not in the FRT-PrPC cells. On the contrary, clathrin-coated pits were found labeled in both type of cells. Arrowheads indicate α PrP gold labelling (10 nm). Bar: a = 238 nm; b = 360 nm; c = 240 nm. Morphometric analysis on 1917 total PrPC gold particles revealed n = 7 in caveolar structures and n = 27 in CCP (clathrin-coated pits, not shown). The remaining 1883 gold particles were distributed on smooth areas of the plasma membrane. PrPC gold particles were counted over 3612 µm of plasma membrane profiles where the number of caveolae and coated pits was comparable (not shown). Note that the percentage of PrPC localized in CCP is similar to wild-type FRT cells (see Figure 4) suggesting that the transfection of caveolin-1 does not affect the surface distribution of PrPC. B) Lysates from FRT-PrPC and FRT-PrPC/cav-1 cells were immunoblotted with α PrP antibody SAF32. M: mature diglycosylated form, I: immature form and U: unglycosylated form. C) Immunofluorescence of PrPC and cav-1 at steady-state shows a similar distribution on the cell surface of FRT-PrPC/cav-1 cells but not after 30 or 60 min of internalization D), suggesting that PrPC endocytosis is not occurring by a caveolae-mediated mechanism (see Methods for experimental procedure). (8.98 MB TIF) [file pone.0005829.s001.tif]

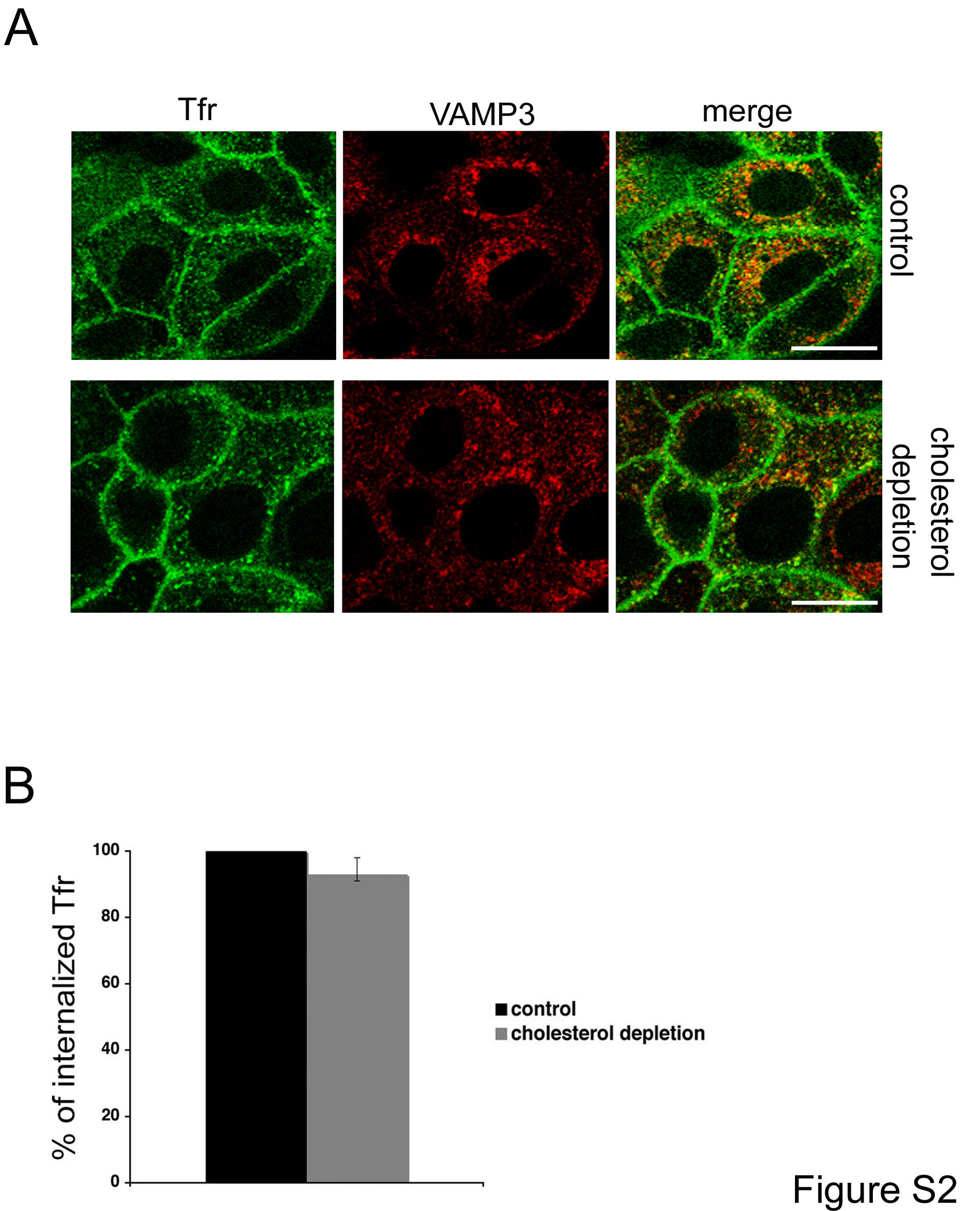

Supplement: Figure S2 — Internalization of Tfr after cholesterol depletion. A) FRT-PrPC cells were grown on coverslipis and incubated for 30 at 37°C in F12 medium with Tfr Alexa 488-conjugated under control or cholesterol depletion conditions. The cells were then fixed with PFA and incubated with αVAMP3 antibody (a marker of the recycling endosomes [83], after permeabilization with saponin. Single and double immunofluorescences (merge) are shown. As shown in panel A, we found that both in control and cholesterol depleted cells Tfr is internalized and partially colocalizes with VAMP3. We measured the internal intensity of fluorescence (IF)/unit of area in optical sections (Z stacks) comprised between the apical and basal surfaces by LSM 510 confocal microscope. A medial optical section is shown. Bar: 10 µm. B) The amount of internalized Tfr in cholesterol depleted cells was expressed as a percentage of the amount of internalized Tfr in control cells, which we set as 100%. Error bars are indicated. The % of internalized Tfr was determined in at least 25 cells from four different experiments under control or cholesterol depletion conditions. The analysis was performed by Image J software. (13.57 MB TIF) [file pone.0005829.s002.tif]

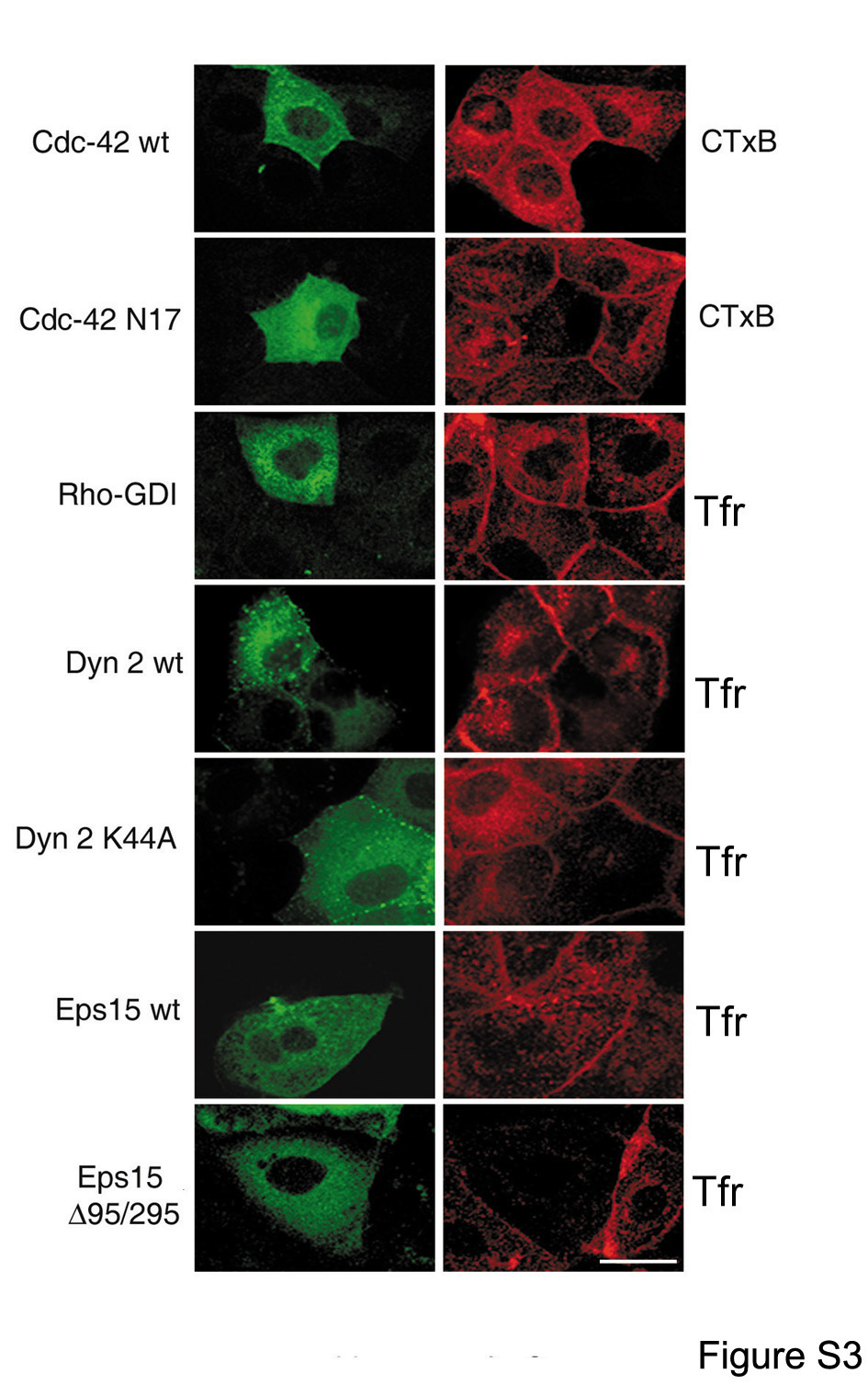

Supplement: Figure S3 — Internalization of CTxB and Tfr upon transient transfection of Cdc- 42, Dyn 2, Eps15 GFP-tagged wt or dominant-negative mutants and of Rho-GDI (His6-tagged form). FRT-PrPC cells were transiently transfected with wild-type (wt) or dominant negative mutant isoforms of GFP-tagged Eps15 (Eps Δ95/295), Cdc-42 (Cdc-42 N17) or Dyn 2 (Dyn 2 K44A). His6-tagged form of Rho-GDI was transiently transfected in FRT-PrPC cells that were processed for immunofluorescence as in Figure 2. CTxB was Alexa-555 conjugated and Tfr was cy3-conjugated. Bar: 10 µm. Data were quantified as described in Table 1. (4.30 MB TIF) [file pone.0005829.s003.tif]

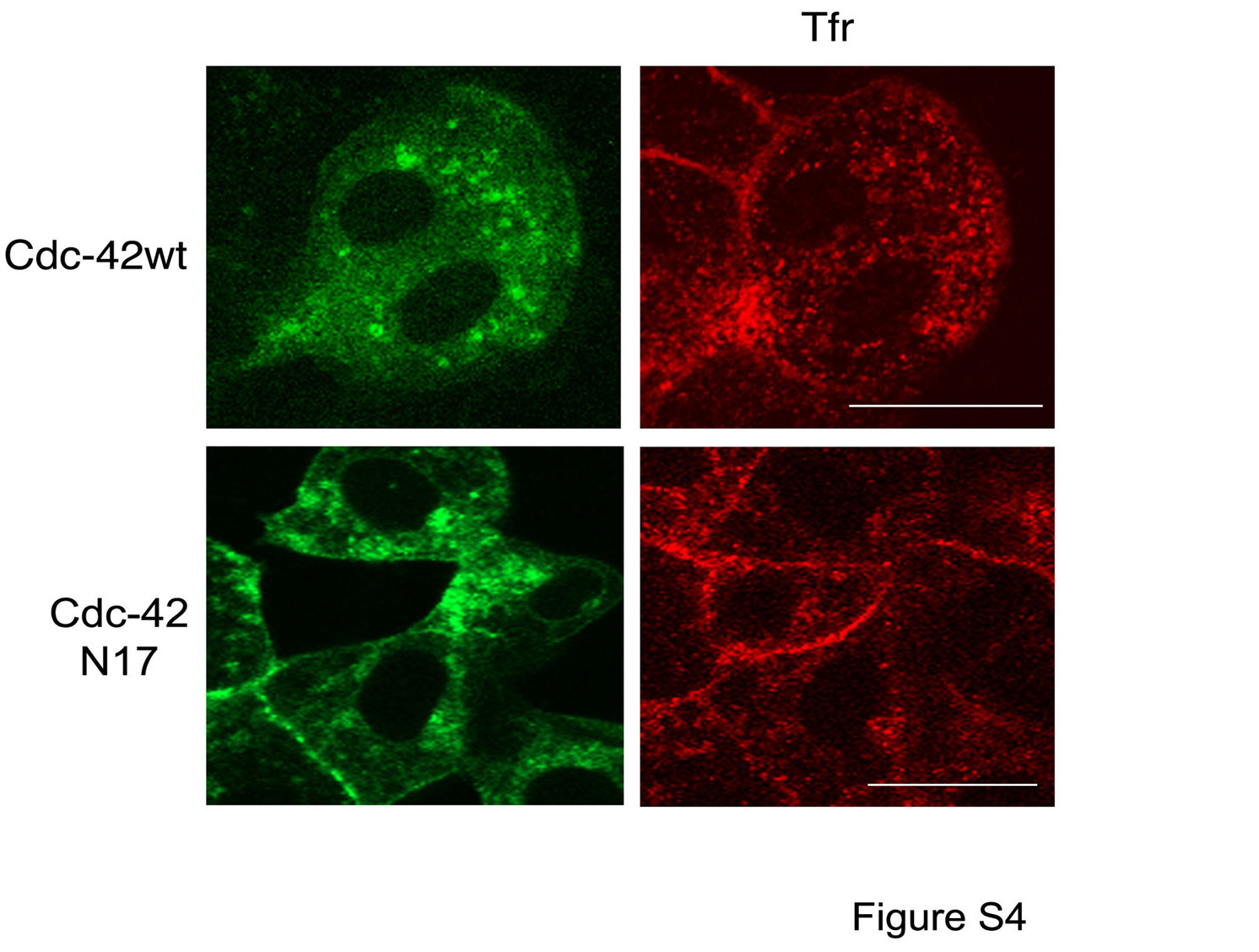

Supplement: Figure S4 — Internalization of Tfr after transient transfection of Cdc-42 wt or N17 mutant. Internalization of Tfr was evaluated after transient transfection of Cdc-42 wt or N17 GFP-tagged isoforms by incubating the cells with cy3-conjugated Tfr internalized for 30 min at 37°C. Bar: 10 µm. Data were quantified as described in Table1. (5.07 MB TIF) [file pone.0005829.s004.tif]

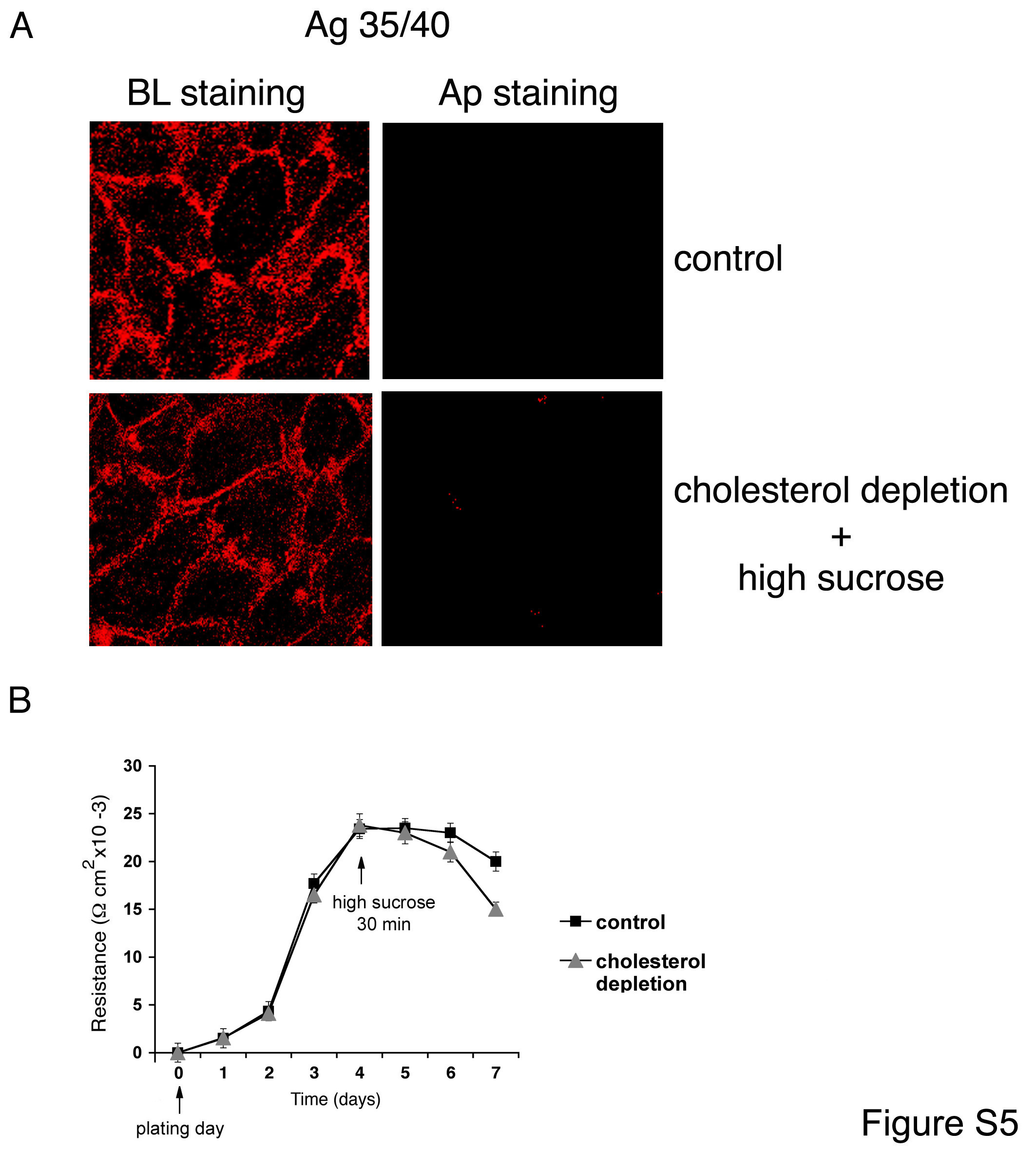

Supplement: Figure S5 — Analysis of Ag35/40 localization and measurement of transepithelial resistance after cholesterol depletion and hypertonic treatment. A) FRT-PrPC cells were grown on transwell permeable filter supports (Costar), in control or cholesterol depleted conditions and incubated for 30 min at 37°C in control or high sucrose medium. Cells where then stained for the basolateral marker Ag35/40 adding a specific antibody either to the apical or basolateral side of the filters. Samples were analyzed with a Zeiss Laser Scanning Confocal Microscope (LSM 510) equipped with a planapo 63× oil-immersion (NA 1.4) objective lens. Bar: 10 µm. B) Transepithelial resistance (TER) was measured for 7 days after plating 2×10^6^ cells on 24 mm diameter transwell filters in control or cholesterol depletion conditions. After 4 days of culture, only cholesterol depleted cells were incubated for 30 min at 37°C in high sucrose medium and TER was measured by the Millicellers apparatus (Millipore). Note that we process the cells at 4 days of culture, when the TER is maximal and that the combined treatment does not affect the monolayer integrity at this time. (15.09 MB TIF) [file pone.0005829.s005.tif]
